# Effects of High Temperature and Heavy Precipitation on Drinking Water Quality and Child Hand Contamination Levels in Rural Kenya

**DOI:** 10.1021/acs.est.2c07284

**Published:** 2023-04-18

**Authors:** Julie E. Powers, Maryanne Mureithi, John Mboya, Jake Campolo, Jenna M. Swarthout, Joseph Pajka, Clair Null, Amy J. Pickering

**Affiliations:** † University of California, Berkeley, Berkeley, California 94704, United States; ‡ Innovations for Poverty Action, Sandalwood Lane, Nairobi 00500, Kenya; § Farmers Business Network, San Carlos, California 94070, United States; ∥ Tufts University, Medford, Massachusetts 02155, United States; ⊥ Mathematica, Washington, D.C. 20002, United States; # Chan Zuckerberg Biohub, San Francisco, California 94158, United States

**Keywords:** Drinking water quality, hands, weather, climate change, pathogens, *E. coli*, low income

## Abstract

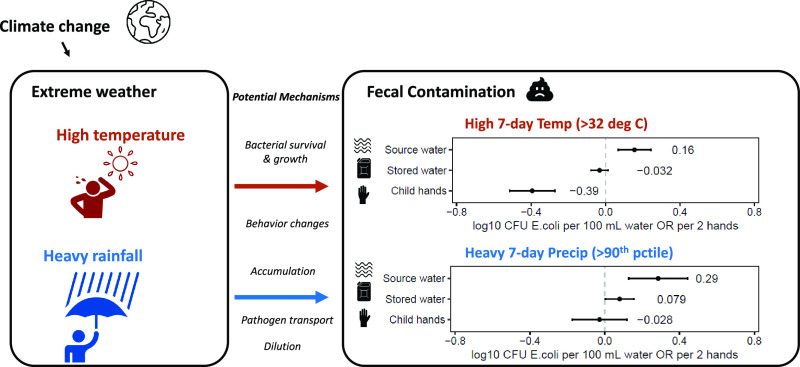

Climate change may impact human health through the influence of weather on environmental transmission of diarrhea. Previous studies have found that high temperatures and heavy precipitation are associated with increased diarrhea prevalence, but the underlying causal mechanisms have not been tested and validated. We linked measurements of *Escherichia coli* in source water (*n* = 1673), stored drinking water (*n* = 9692), and hand rinses from children <2 years old (*n* = 2634) with publicly available gridded temperature and precipitation data (at ≤0.2 degree spatial resolution and daily temporal resolution) by the GPS coordinates and date of sample collection. Measurements were collected over a 3-year period across a 2500 km^2^ area in rural Kenya. In drinking water sources, high 7-day temperature was associated with a 0.16 increase in log_10_*E. coli* levels (*p* < 0.001, 95% CI: 0.07, 0.24), while heavy 7-day total precipitation was associated with a 0.29 increase in log_10_*E. coli* levels (*p* < 0.001, 95% CI: 0.13, 0.44). In household stored drinking water, heavy 7-day precipitation was associated with a 0.079 increase in log_10_*E. coli* levels (*p* = 0.042, 95% CI: 0.07, 0.24). Heavy precipitation did not increase *E. coli* levels among respondents who treated their water, suggesting that water treatment can mitigate effects on water quality. On child hands, high 7-day temperature was associated with a 0.39 decrease in log_10_*E. coli* levels (*p* < 0.001, 95% CI: −0.52, −0.27). Our findings provide insight on how climate change could impact environmental transmission of bacterial pathogens in Kenya. We suggest water treatment is especially important after heavy precipitation (particularly when preceded by dry periods) and high temperatures.

## Abstract 2

Weather extremes may impact transmission of fecally transmitted pathogens. In Kenya, we showed that high temperature and heavy precipitation increased fecal bacteria (*E. coli*) levels in drinking water, while high temperature decreased levels on child hands.

## Introduction

In 2019, diarrhea was the ninth leading cause of death in all ages of people and the third leading cause of death in children under five.^1^ Diarrhea also contributes to malnutrition,^2−5^ stunting,^6−12^ and cognitive impairment^6,7,13−20^ that could extend into adulthood.^7,21^ Diarrhea is caused by enteric pathogen infections (bacterial, viral, or parasitic^22^) that are transmitted via the fecal–oral route: contaminated feces from an infected human or animal spread through environmental pathways (fluids, fingers, fields, food, fomites, flies) are ingested by another person.^22−26^ Progress has been made in reducing the global burden of diarrhea: Between 2005 and 2015, under-5 deaths due to diarrhea per population decreased by 39.2%, and diarrhea incidence decreased by 10.4%.^27^ Diarrhea incidence has not decreased as quickly as diarrhea-associated mortality, suggesting that improved access to treatment may be largely responsible for the reductions in mortality.

Climate change is expected to shift weather patterns globally, including in Sub-Saharan Africa, where the burden of diarrhea-related mortality is already very high.^1^ On a global level, as mean surface temperature rises, extreme precipitation events are projected to become more frequent and intense, and heat waves are projected to become more frequent and longer in duration.^28^ In East Africa, mean annual temperature is expected to increase 2–4 °C by 2050.^29^ Precipitation projections vary widely: some models predict a potential increase of 2–4 extreme precipitation events annually in East Africa (Kenya and Tanzania)^30^ while others predict an increase in intensity and density of extreme precipitation events, but not a change in the actual number of events.^31^

Extreme weather associated with climate change could increase the global burden of diarrhea because temperature^32−38^ and heavy rainfall^35,38−40^ are positively associated with diarrhea. A systematic review by Levy et al. found significant positive relationships between temperature and diarrhea (observed in 65% of quantitative studies, *n* = 82) and heavy rainfall and diarrhea (observed in 71% of quantitative studies, *n* = 14).^41^ Some studies found that the association between rainfall and diarrhea only holds following prolonged dry periods.^39,42,43^ Notably, of the 55 temperature and heavy rainfall studies identified by Levy et al., only nine were conducted in Sub-Saharan Africa.^41,44^

One pathway by which weather may affect diarrhea is via increased contamination of drinking water. Heavy rainfall may cause surface runoff and flooding, potentially transporting feces and contaminating drinking water sources. However, heavy rainfall could also dilute the concentration of fecal matter in drinking water sources. High temperatures may influence pathogen survival in the environment, but the direction of the effect is unclear: pathogens may die off at a faster rate under high temperature conditions, but growth could also accelerate if sufficient nutrients are present.^45,46^ A few recent studies found that heavy rainfall was associated with increased *Escherichia coli* (fecal indicator bacteria) levels in drinking water sources^44,47−50^ and household stored water^47,48,50^ in locations in Bangladesh,^48,49^ Burkina Faso,^44^ Nepal,^48^ and Tanzania.^47,48,50^ Higher temperatures increased *E. coli* levels in Bangladesh and Nepal, but decreased *E. coli* levels in Tanzania.^48^ This variation by location suggests that the effects of weather on water quality are highly context specific and underscores the need for evidence from additional locations.

In addition to physical and biological mechanisms, temperature and precipitation extremes could also lead to community or household-level behavioral changes that influence water quality.^48^ At the community level, agricultural activities such as application of animal feces as fertilizer may be correlated with temperature and precipitation. Planting occurs twice per year for many common crops in Kenya’s Kakamega and Bungoma counties (located in Food and Agriculture Organization’s Upper and Lower Midland Zones): once in February to March and again in August to October.^51^ February to March is typically warmer than average, and both planting periods directly precede the rainy seasons, which occur from March to June and from October to December.^52^ Effects on household stored water could differ from drinking water sources if water-related behaviors are associated with weather. At the household level, it is common to use multiple water sources for domestic activities.^53,54^ If heavy rainfall or high temperatures lead to perceived changes in water quality (e.g., color or turbidity), households may decide to switch sources or treat their water.

Weather could affect hand contamination through behavioral changes or through other mechanisms. Sweat secretions include peptides that have antimicrobial activity against bacteria including *E. coli*.^55−57^ Increased sweating during hot weather could increase bacteria die-off. There could also be lower transfer efficiency from fomites to hands at low relative humidity (which is inversely related to temperature). Lopez et al. found that *E. coli* had a lower transfer efficiency from fomites to fingers at low relative humidity.^58^ Temperature may also influence handwashing behavior: Charles et al. found that hands appeared dirtier during cool weather in Nepal and that respondents were less likely to wash their hands during cool weather in Bangladesh.^48^ Heavy rain could lead to higher water availability on premises (e.g., via rainwater collection), which has been linked to improved hand hygiene.^59^ Hand contamination is positively associated with stored drinking water contamination, suggesting that fecal contamination on hands could contribute to fecal contamination in stored water.^60,61^ The effects of weather on hands and stored water could be related. We are not aware of any studies that have examined the effects of weather on microbial hand contamination.

In this study, we examine associations between recent weather (heavy precipitation and high temperature) and environmental *E. coli* contamination (source water, stored water, and child hands) in Kenyan households. We also examine effect modification by water treatment, source type, water storage container, and low long-term precipitation. Finally, we investigate how recent weather affects behaviors that could influence water and hand contamination.

## Materials and Methods

### Data Sources

**Figure 1:**
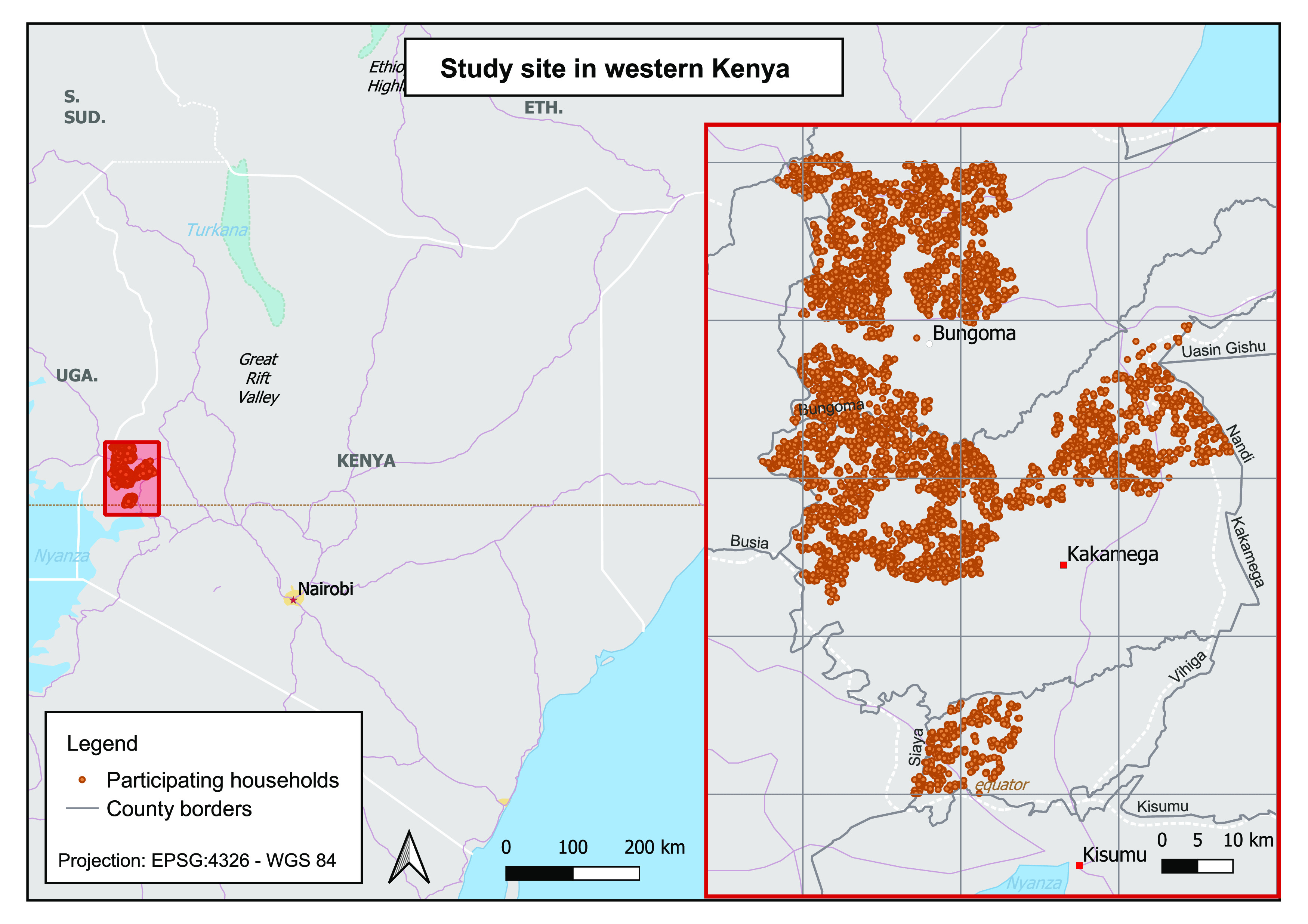
Study site (2500 km^2^) in western Kenya. Participating households (*n* = 5,761) are plotted.

We leveraged environmental *E. coli* contamination data from the WASH Benefits Study in western Kenya (Figure 1), a multiyear randomized controlled trial that enrolled pregnant women and studied the effects of water, sanitation, hygiene, and nutrition interventions on diarrhea and growth in children during their first two years of life.^62,63^ The study design has been published elsewhere.^62^ Investigators designed the trial with a control arm (C) and six intervention arms: water treatment (W); sanitation (S); handwashing with soap (H); combined water, sanitation, and handwashing (WSH); nutrition (N); and combined water, sanitation, handwashing, and nutrition (WSHN) (see Supplementary Methods).

WASH Benefits visited households prior to intervention delivery (baseline, 2012–2014) and approximately one (midline) and two years (endline) after intervention delivery. Field staff conducted surveys at all time points. At midline and endline, field staff observed if respondents washed their hands during the visit. Investigators assessed environmental contamination in a subset of households: Trained field staff collected source water samples (*n* = 1673) only at baseline; stored water samples from the C/N, WSH/WSHN, W, and H arms at baseline (*n* = 5761), midline (*n* = 1577), and endline (*n* = 2354); and child hand rinse samples from the C/N and WSH/WSHN arms at midline (*n* = 1026) and endline (*n* = 1646). These staff were trained to collect water and hand rinse samples using a sterile technique. Observations from the C and N arms (C/N) and observations from the WSH and WSHN arms (WSH/WSHN) were grouped because nutrition was not expected to impact environmental contamination. GPS coordinates were collected for each water source and for each household. For stored water collection, field staff asked respondents to show them what they would use if their child 0–3 years old wanted a drink of water. Field staff also sampled the water source that the household reported collecting from if it was within the same village. All water samples were collected as 150 mL samples in sterile Whirlpak bags. If the respondent reported adding chlorine to the stored water, study staff added sodium thiosulfate to neutralize chlorine residual and measured free chlorine residual using the Hach Color Wheel (detectable if >0 mg/L). Child hand rinse samples were collected by filling a Whirlpak bag with 250 mL of clean distilled water, placing the index child’s hands in the bag one at a time, massaging the hand, and shaking the hand. More details on this method have been published elsewhere.^60,64,65^

All samples were transported to the field lab on ice and processed the same day of collection. Laboratory technicians analyzed all environmental contamination samples by membrane filtration with MI media (BD, United States) to detect *E. coli* and incubated at 35 °C for 20 h following U.S. Environmental Protection Agency approved method 1604 (detection limit of 1 CFU per 100 mL water or 1 CFU per 2 child hands).^66^*E. coli* is commonly used as an indicator of fecal contamination^67^ and was assessed rather than enteric pathogens due to budget constraints.

We paired gridded meteorological data with point household observations using Google Earth Engine Code Editor, a web-based integrated development environment for the Google Earth Engine JavaScript API. We extracted temperature and precipitation data from the following publicly available gridded sources using the sample collection location GPS coordinates and date of collection:

- *Precipitation*: Climate Hazards Group InfraRed Precipitation with Station data (CHIRPS) is a quasi-global rainfall time series data set spanning 1981 to present. Daily precipitation is available at 0.05 degree resolution, corresponding to an area approximately 5.6 km by 5.6 km.^68^
- *Temperature*: National Centers for Environmental Prediction (NCEP) Climate Forecast System (CFS) spans 1979 to present. Maximum land surface temperature is available for every 6 h at 0.2 degree resolution, which corresponds to approximately 22.3 km by 22.3 km.^69^

### Primary Analysis

We performed data cleaning and analysis in Stata/MP 16.1 and RStudio version 2022.07.0. Missing samples were excluded. For our primary analysis, we used multivariate ordinary least-squares (OLS) linear regression to examine the combined effect of heavy precipitation and high temperature on log_10_-transformed *E. coli* levels in source water, stored water, and on child hands. Samples with uncountable *E. coli* colonies (e.g., because the plate was smudged) were considered positive but excluded from regression analyses of continuous *E. coli* levels. Samples with colonies that were too numerous to count (>500 CFUs) were approximated as 500 CFU; samples under the detection limit were included as 0.5 CFU/100 mL prior to log10 transformation. We controlled for WASH Benefits treatment arm in all models. We clustered standard errors at the cluster level. Hypotheses and statistical analysis methods are listed in Supplementary Table 1.

Studies examining links between weather and water quality and/or diarrhea have defined temperature and precipitation exposures in several ways. A systematic review^41^ of weather and diarrhea risk found heterogeneous exposure definitions including daily,^32^ weekly,^70^ biweekly,^71^ and monthly^34−36,72−75^ time scales; average,^32,34,36^ maximum,^35,70−72,75,76^ and minimum temperature;^71,72,75,77^ 90th percentile,^39,42^ 95th percentile,^78^ and other thresholds of heavy precipitation.^35,37,79^ In a study of weather and water contamination, Guo et al. included eight potential weather predictors of *E. coli* levels and used Bayesian hierarchical modeling to find the best combination of predictors.^47^ Robert et al. found both daily and weekly precipitations were correlated with *E. coli* levels, but the correlation with weekly precipitation was slightly stronger.^44^ Charles et al. examined extreme events using daily precipitation (90th, 95th, and 99th percentiles), daily minimum temperature (10th, 5th, and 1st percentiles), and daily maximum temperature (90th, 95th, and 99th percentiles).^48^

In the absence of clear consensus on exposure definition in the diarrhea and water quality literature, we selected exposures based on our hypothesized mechanisms and ease of interpretability. We selected exposures at the weekly level based on the assumption that *E. coli* survival is at a similar time scale. We did not use daily exposures because we expected that there may be lagged effects (e.g., due to households storing water for multiple days) and because there could be cumulative effects (e.g., due to multiple heavy rainfall events in the same week). We selected thresholds because we were primarily interested in the effects of extreme weather and because they may be easier for policymakers and users to interpret. We defined heavy precipitation as sample collection dates on which the total precipitation in the preceding 7 days exceeded the 90th percentile of our data (72.7 mm). The 90th percentile is commonly used in the climate projection literature^80^ and thus allows results to be translatable. We defined high temperature as sample collection dates on which the mean of the daily maximum in the preceding 7 days exceeded 32 °C, a commonly used threshold for severe heat strain for humans.^81,82^ We used daily maximums because we were interested in the effects of extreme weather. We excluded the day of sample collection because samples were collected throughout the day and thus were not exposed to the temperature and precipitation conditions during the entire 24-h period.

### Sensitivity Analysis

Because the results may be sensitive to the chosen predictor variable definitions (7-day period, choice of “heavy/high” thresholds), we performed several sensitivity analyses with varied specifications to assess if the relationships are robust. We repeated the analysis using (1) 5-day time periods rather than 7-day time periods, (2) absolute precipitation and temperature rather than thresholds, (3) the 90th percentile of 7-day mean max temperature (35.8 °C) as a threshold for “high temperature”, rather than 32 °C, and (4) heavy 1-day precipitation (exceeds 90th percentile, 15.5 mm) during any 1-day period in the previous 7 days (as in Carlton et al.^39^) rather than total 7-day precipitation.

### Interaction Analysis

We assessed whether the joint effects of heavy precipitation and high temperature differ from the sum of the individual effects by repeating the above primary analysis with an interaction term (heavy 7-day precipitation × high 7-day temperature). We computed the joint effects of heavy precipitation and high temperature by adding the independent and interaction effects. We did not include interaction effects in the primary analysis because temperature and precipitation are often closely correlated.^83^

### Effect Modification: 8-Week Rainfall

We considered 8-week precipitation as a potential effect modifier because some studies have found that the association between rainfall and diarrheal illness only holds following prolonged dry periods.^39,42,43^ We calculated 8-week precipitation tertiles as done in Carlton et al. and Deshpande et al.^39,42^ We repeated the analysis stratified in two subgroups: low precipitation (0th to 33rd percentile, 23–222 mm) compared to moderate or high precipitation (>33rd percentile, 223–760 mm).

### Effect Modification: Water Treatment

We hypothesized that water treatment may mitigate the effects of weather on water quality. For this reason, we examined self-reported water treatment (any method) and confirmed chlorine water treatment (detectable free chlorine residual >0 mg/L) as effect modifiers.

### Effect Modification: Water Storage Container

We examined water storage container type as an effect modifier (for container types with >100 observations) because the storage container can affect recontamination risk^84^ and the storage temperature of the water.^85^

### Effect Modification: Source type

We considered improved source versus unimproved source as an effect modifier because we hypothesized that water collected from an unimproved source may be more susceptible to contamination during heavy precipitation events than water collected from an improved source.

In a separate analysis, we also considered specific source type as an effect modifier (for source types with >100 observations) because mechanisms may differ by source type. For example, surface water sources (streams, rivers, lakes, ponds) are generally more open and may be more exposed to sunlight during hot weather (potentially inactivating bacteria) compared with other source types. In addition to conducting stratified analyses, we tested for statistical significance of effect modifiers by including an interaction term in our multivariate models.

### Behavior Analysis

Because respondents may react to changes in weather, we examined effects of heavy 7-day precipitation (>90th percentile of our data, 72.7 mm) and high 7-day mean maximum temperature (mean of daily maximums >32 °C) on several behavioral measures: collection from an improved source, what source type water was collected from, water treatment, what treatment methods were used, how long the water was stored for, and whether the respondent was observed washing their hands during the visit. We used multivariate modified Poisson regression^86^ for binary outcomes (improved source, source type, treatment, treatment method, and handwashing). This model specification is often used with binary outcomes and has been shown to perform well.^87−89^ It also has the advantage of generating prevalence ratios instead of odds ratios, which are easier to interpret.^87,88^ We used multivariate OLS regression for continuous outcomes (water storage time). We controlled for treatment status in all models.

## Results

As previously reported,^89^*E. coli* prevalence was high (percent of samples positive for *E. coli* was >90%) among all water source types and on child hands (Table 1). *E. coli* levels (CFU/100 mL) were lower in household stored water (median = 29 CFU/100 mL) than in water sources (median = 69 CFU/100 mL). The median *E. coli* level on child hands was 37 CFU/100 mL.

**Table 1:**
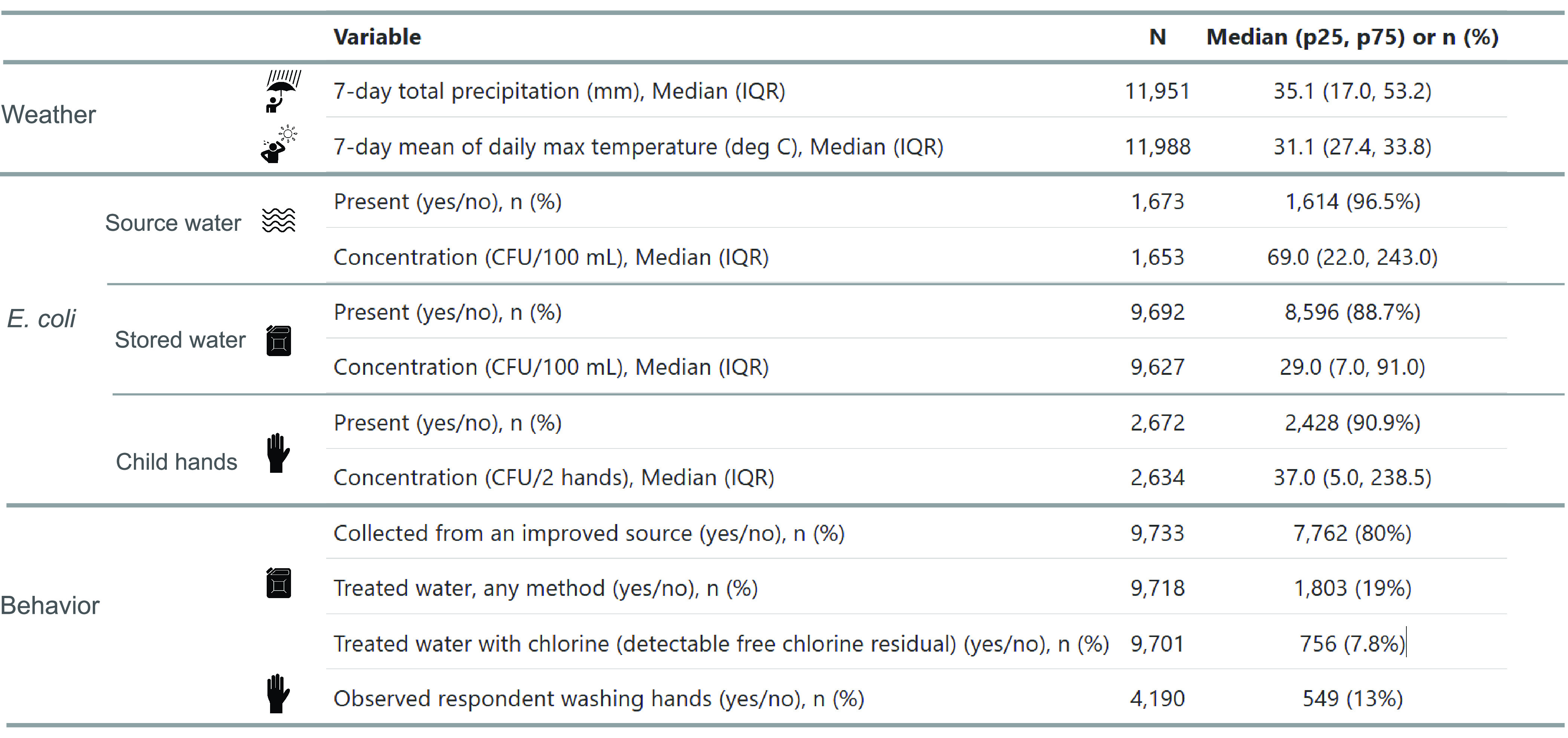
Descriptive Statistics^a^

a Sample size (*n*) is shown for all variables. Median, 25th percentile, and 75th percentile are shown for continuous variables. Prevalence of yes responses (number and percent) is shown for binary variables. Sample sizes are smaller for *E. coli* concentration variables than for *E. coli* presence variables because a small number of plates with *E. coli* colonies was uncountable (*n* = 20 for source water, *n* = 65 for stored water, and *n* = 38 for child hands).

### Primary Analysis

**Figure 2:**
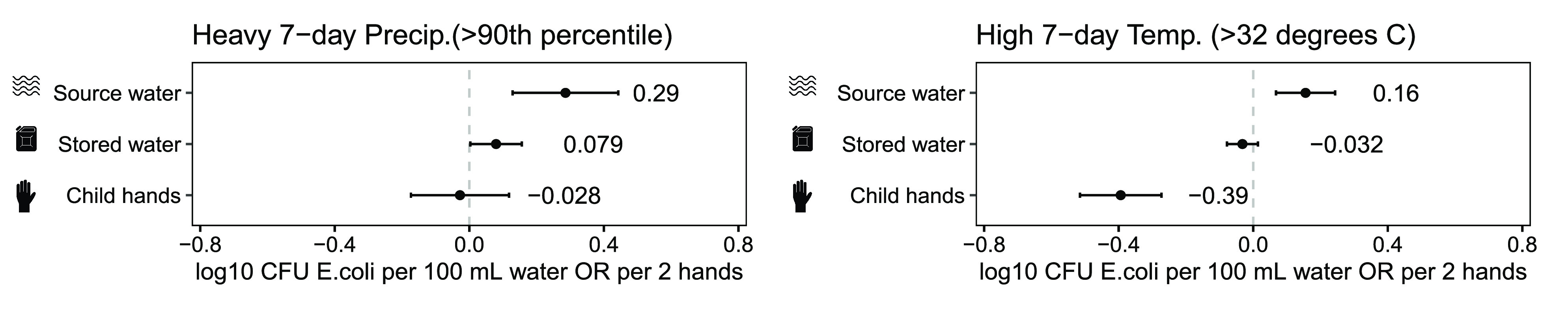
Associations between heavy 7-day precipitation (left), high 7-day temperature (right), and *E. coli* levels in source water, stored water, and child hands. Point estimates are plotted and labeled. Units are log_10_ CFU *E. coli* per 100 mL for source water and stored water and per 2 hands for child hands. Error bars show 95% confidence intervals.

High temperature and heavy total precipitation during the week before sample collection were significantly associated with environmental *E. coli* levels (Figure 2). In water sources, heavy precipitation (>90th percentile) was associated with a 0.29 increase in log_10_ CFU *E. coli* per 100 mL water (*p* < 0.001, 95% CI: 0.13, 0.44), and high temperature (mean >32 °C) was associated with a 0.16 increase in log_10_ CFU *E. coli* per 100 mL water (*p* < 0.001, 95% CI: 0.07, 0.24). In household stored water, heavy precipitation was associated with a 0.079 increase in log_10_ CFU *E. coli* per 100 mL water (p = 0.042, 95% CI: 0.003, 0.16). High temperature was not significantly (*p* > 0.05) associated with *E. coli* levels in household stored water.

Heavy precipitation was not significantly associated with *E. coli* levels on child hands. However, high temperature was significantly associated with a 0.39 decrease in log_10_*E. coli* CFU per two hands (*p* < 0.001, 95% CI: −0.52, −0.27)

### Sensitivity Analysis

Results were consistent across multiple sensitivity analyses (see Supplementary Figures 1–4).

### Interaction Analysis

We observed a significant negative interaction (−0.36 log_10_ CFU *E. coli* per 100 mL water, 95% CI: −0.67, −0.04) between the effects of heavy precipitation and high temperature on *E. coli* levels in water sources (*p* = 0.027 on the interaction term, Supplementary Figure 5). While heavy precipitation alone was associated with a 0.45 increase in log_10_ CFU *E. coli* per 100 mL water (95% CI: 0.29, 0.61) and high temperature alone was associated with a 0.17 increase in log_10_ CFU *E. coli* per 100 mL water (95% CI: 0.07, 0.26), the joint effect of heavy precipitation and high temperature was a 0.26 increase in log_10_ CFU *E. coli* per 100 mL water.

### Effect Modification: 8-Week Rainfall

**Figure 3:**
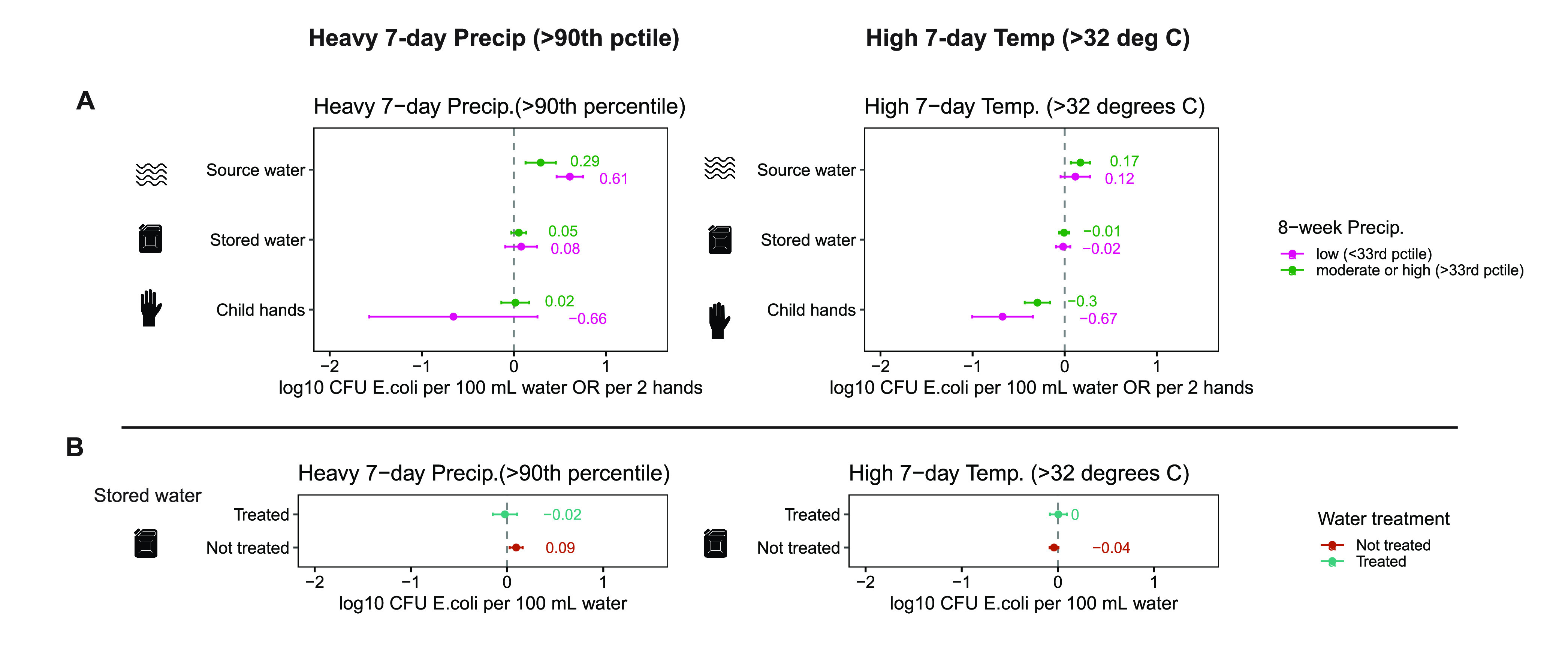
Effect modification by (A) low long-term (8-week) precipitation and (B) self-reported water treatment. Associations between heavy 7-day precipitation (left), high 7-day temperature (right), and *E. coli* levels in source water, stored water, and child hands. Point estimates are plotted and labeled. Units are log_10_ CFU *E. coli* per 100 mL. Error bars show 95% confidence intervals. (A) Results are stratified by low (0th to 33rd percentile) vs moderate or high (>33rd percentile) 8-week rainfall. (B) Results are stratified by treated vs not treated.

Low long-term precipitation modified the effect of heavy precipitation on *E. coli* levels in water sources (*p* = 0.004 on interaction term, Supplementary Table 2), with a larger increase (0.61 log_10_ CFU *E. coli* per 100 mL) after low 8-week rainfall compared to moderate or high 8-week rainfall (Figure 3A). Low long-term precipitation also modified the effect of high temperature on *E. coli* levels on child hands (*p* = 0.045 on interaction term, Supplementary Table 2), with a larger reduction (−0.67 log_10_ CFU *E. coli* per 100 mL) after low 8-week rainfall (Figure 3A).

### Effect Modification: Water treatment

Water treatment modified the effect of heavy precipitation on *E. coli* levels in stored water (*p* < 0.001 on interaction term, Supplementary Table 3). While heavy precipitation was associated with increased *E. coli* levels (0.094 log_10_ CFU *E. coli* per 100 mL) in stored water from households who did not treat their water, this relationship did not hold among households who treated their water (Figure 3B).

Chlorine water treatment (confirmed by a chlorine residual test) did not modify the effects of heavy precipitation or high temperature on *E. coli* levels in stored water (Supplementary Figure 7, Supplementary Table 4); chlorine-treated water was a relatively small subset of treated water (*n* = 756).

### Effect Modification: Water storage container

Water storage container type modified the effects of high temperature on *E. coli* levels in stored water (Supplementary Figure 8, Supplementary Table 7). As shown above, high temperature was weakly associated with a slight decline in *E. coli* levels in stored water overall. However, among water collected from clay pots, high temperature was associated with a larger and statistically significant decline in *E. coli* levels (0.13 log_10_ CFU *E. coli* per 100 mL, *p* = 0.016 on interaction term).

### Effect Modification: Improved Source

Heavy precipitation was associated with a larger increase in *E. coli* levels in unimproved sources (0.42 log_10_ CFU *E. coli* per 100 mL, *p* = 0.026 on interaction term, Supplementary Figure 6A, Supplementary Table 5) compared to water from improved sources.

### Behavior

**Figure 4:**
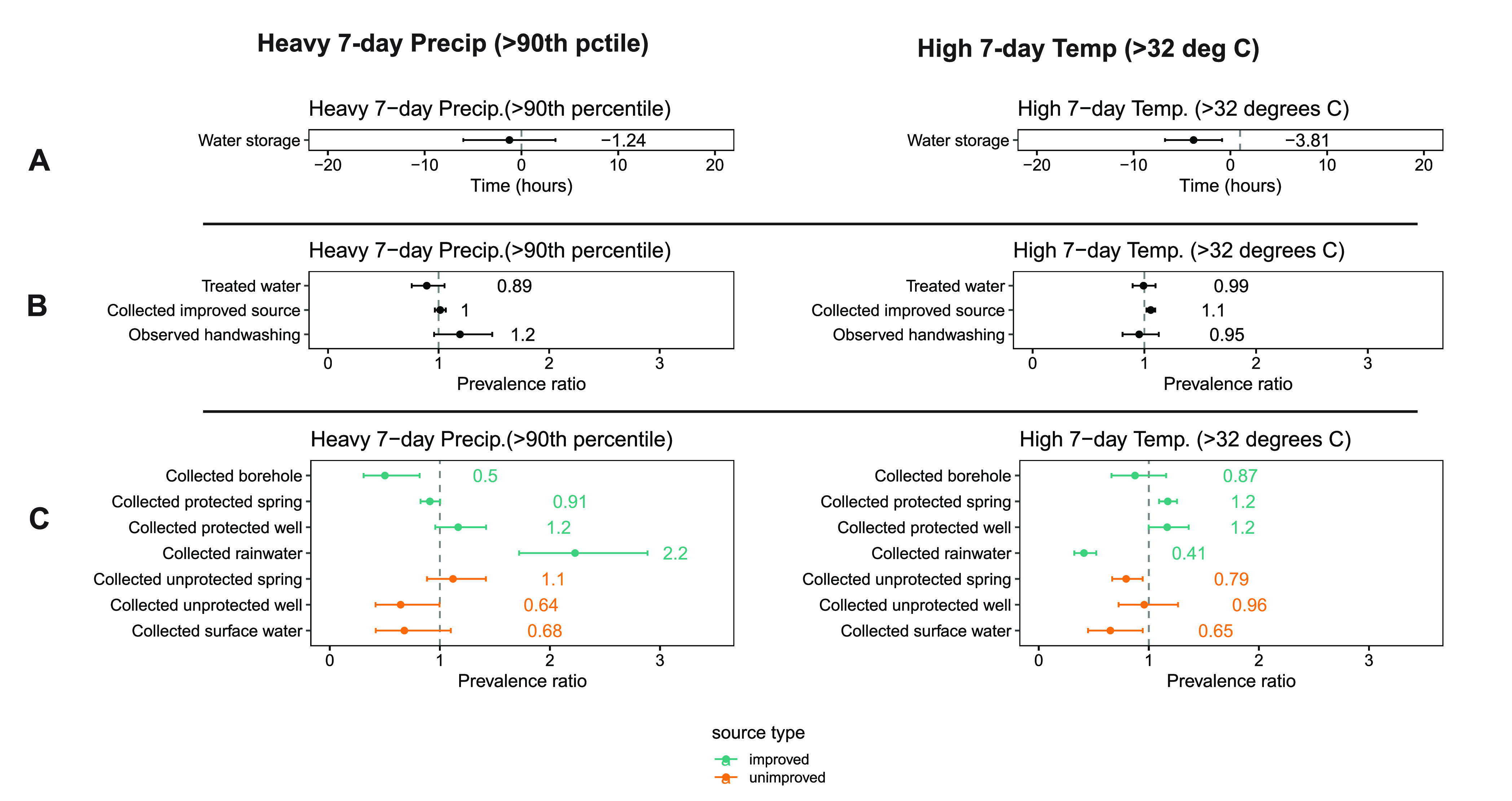
Associations between heavy 7-day precipitation (left), high 7-day temperature (right), and reported stored water behaviors: water storage time (A), water treatment and collection from an improved source (B), and source type that the respondent reported collecting water from (C). Water storage time in hours is plotted and labeled for (A). Prevalence ratios are plotted and labeled for (B) and (C). Error bars in all panels show 95% confidence intervals.

We found evidence that households altered water and handwashing behaviors in response to the weather. Households were more likely to collect water from an improved source after high 7-day temperature (prevalence ratio = 1.1, *p* = 0.003, Figure 4B), driven predominantly by an increased likelihood of collection from a protected spring or protected well (improved) and decreased likelihood of collection from an unprotected spring or source water (unimproved) (Figure 4C). High 7-day temperature was also associated with shorter water storage time (mean difference = 3.81 h, *p* = 0.01, Figure 4A). High temperature was not significantly associated with respondent handwashing (Figure 4B).

Heavy precipitation was not significantly associated with the decision to treat water or collect from an improved source (Figure 4B). However, households were more likely to collect rainwater after heavy rain (prevalence ratio = 2.2, *p* < 0.001, Figure 4C). Respondents were more likely to collect rainwater after heavy 7-day precipitation (prevalence ratio = 2.2, *p* < 0.001) and less likely to collect rainwater after high 7-day temperature (prevalence ratio = 0.41, *p* < 0.001, Figure 4C). Heavy 7-day precipitation was weakly associated with increased handwashing (prevalence ratio = 1.2, *p* = 0.18, Figure 4B).

## Discussion

We found that heavy precipitation and high temperature had meaningful and statistically significant effects on *E. coli* levels in water sources, stored water, and child hands. These effects were consistent across multiple sensitivity analyses, suggesting that the effects were true and not sensitive to the choice of model specification.

In water sources, heavy rainfall increased *E. coli* levels, perhaps by transporting feces via increased runoff and flooding. This finding is consistent with studies in other locations.^44,47,48^ The effect size (increase of 0.29 log_10_ CFU *E. coli* per 100 mL water) was similar in magnitude to the effect associated with using an improved source (reduction of 0.33 log_10_ CFU *E. coli* per 100 mL water), indicating that the effect is meaningful. The effect was larger following low 8-week rainfall (0.69 log_10_ CFU *E. coli* per 100 mL water). We hypothesize that this could be because long dry periods may have allowed fecal contamination to accumulate in the environment. The effect was larger in unimproved sources compared to improved sources, likely because unimproved sources are at higher risk for contamination via runoff or flooding. This finding is consistent with two of three previous studies.^47,48,50^

Heavy rainfall also increased *E. coli* levels in household stored water (0.079 increase in log_10_ CFU *E. coli* per 100 mL water). The effect size is about half of the magnitude associated with collection of stored water from an improved source (0.19 reduction in log_10_ CFU *E. coli* per 100 mL water). As a reference, a change of one log_10_ CFU *E. coli* per 100 mL water represents a shift in WHO risk classification (0 CFU per 100 mL is in conformity, 1–10 CFU per 100 mL is low risk, 10–100 CFU per 100 mL is intermediate risk, 100–1000 CFU per 100 mL is high risk, and >1000 CFU per 100 mL is very high risk).^90^ The effect of heavy rainfall is small relative to the one log_10_ change associated with a change in risk classification. The effect of temperature was smaller in household stored water compared with in water sources (consistent with other studies^47,48^), potentially because stored water quality is more complex and impacted by numerous household-level behavioral choices. For example, we found that households were more likely to collect rainwater (improved source) after heavy precipitation. This could diminish the effects of elevated *E. coli* levels in water sources after heavy precipitation because the mechanisms by which heavy precipitation affects water quality in other source types (e.g., runoff) may not apply to rainwater collection (Supplementary Figure 6). Although rainwater collection was associated with improved water quality in our data (reduction of 0.09 log_10_ CFU *E. coli* per 100 mL), roof-harvested rainwater is not always free from microbial^91,92^ and chemical^91,93^ contaminations both because contaminants from the atmosphere may be present in rainwater and because contaminants may accumulate on the roof. The effect on stored water could also be smaller because *E. coli* levels were generally lower in household stored water than in water sources, potentially because some households (19%) reported treating their water. Notably, heavy rainfall did not increase *E. coli* levels among the subset of respondents who reported treating their water, suggesting that water treatment can mitigate effects on water quality. We observed a negative interaction between heavy precipitation and high temperature in water sources: though both were significantly associated with increased *E. coli* levels, the joint effect was smaller than would be expected by adding their independent effects.

High temperatures increased *E. coli* levels in water sources (0.16 log_10_ CFU *E. coli* per 100 mL) but may have slightly decreased *E. coli* levels in stored water (reduction of 0.03 log_10_ CFU *E. coli* per 100 mL, 95% CI: −0.08, 0.01). This could be partially explained by lower hand contamination during high temperatures, which may have reduced contamination of household stored drinking water when household members interact with stored water. Others have found that hand contamination and stored water contamination are closely correlated.^60,61^ The observed increase in *E. coli* levels in water sources during high temperatures suggests that high temperature may have increased bacterial growth in water more so than die-off. These effects may be attenuated if water is cooled during storage. We found that high temperatures decreased *E. coli* levels in water stored in clay pots but did not significantly change *E. coli* levels in water stored in jerry cans or plastic buckets. Others have shown that water stored in clay pots stays at a lower temperature than in plastic and metal containers due to the evaporative cooling properties of clay,^85,94^ potentially reducing bacterial growth. Some natural clays have antibacterial properties^95^ which could have also increased die-off during storage. High temperature was also associated with shorter water storage time, perhaps because households drink^96,97^ or use^98,99^ more water during hot weather. Because water storage time can make water more prone to recontamination,^100^ shorter storage time could also mitigate the effects of high temperature. Finally, high temperatures may be correlated with agricultural activities such as manure application (planting occurs at relatively warm times of year^51^), which could have led to increased *E. coli* levels in water sources. We found that households were more likely to collect water from an improved source after high temperatures. This could be due to changes in availability (e.g., some sources may dry up during hot weather) or because respondents choose different water sources based on perceived changes in quality (e.g., color, turbidity, taste, knowledge of ongoing agricultural activities). Increased collection from an improved source may have mitigated the effect of elevated *E. coli* levels in water sources after high 7-day temperature because collection from an improved source was associated with improved water quality (reduction of 0.19 log_10_ CFU *E. coli* per 100 mL in our data). Charles et al. also observed behavioral changes in response to the weather, including more frequent water collection during hot weather in Tanzania and increased water treatment during the dry season in Bangladesh.^48^

As climate change progresses and extreme precipitation and temperatures are more common, we may see higher *E. coli* levels in drinking water sources, particularly among sources that are unimproved. Improved sources are thus likely to be more resilient to extreme weather but will still be affected. We also expect to see higher *E. coli* levels in household stored water, though to a lesser extent than in water sources. We show that individuals react to weather in ways that may mitigate effects on household stored water. Water should always be treated and others have shown that strict adherence is necessary to reap all potential health benefits.^101^ However, in absence of universal treatment, water treatment may be particularly important after periods of heavy rainfall (particularly when preceded by a dry period) or high temperatures. Programs with limited resources could promote or implement water treatment after heavy rain or high temperatures. Water treatment uptake was relatively low (19%) in the study area. These results also underscore the need for scaling of water treatment solutions that minimize the need for individual-level behavior change. When households are required to bear the time and financial burden of water treatment, the poorest and most vulnerable households are likely to be disproportionately affected by climate change.

In this first study of weather and hand contamination, high temperature decreased *E. coli* levels on child hands. There are a few potential explanations for this finding. Heat and sunlight may have increased *E. coli* die-off. Increased sweating during hot weather may have increased *E. coli* die-off because sweat secretions contain antimicrobial peptides.^55−57^ Low relative humidity (inversely related to temperature) may have reduced bacterial transfer from contaminated fomites (e.g., floor, toys) to child hands.^58^ Temperature is unlikely to influence hand washing effectiveness^102−104^ or hand rinse sample bacterial yield,^105^ but it could impact hand washing frequency. Charles et al. found that handwashing decreased during cool weather in Bangladesh.^48^ However, we did not observe an association between temperature and handwashing in our data. Bathing may also increase during warm weather because children and their caregivers may use water to cool down when it is hot. Traore et al. found that children in Burkina Faso swam and mothers bathed under-five children more frequently during a hot period compared with a cold period.^106^ Increased bathing or other water contact could have had co-benefits for hand hygiene: Pickering et al. found that bathing (of self-or child) decreased *E. coli* levels on mothers’ hands.^107^ Children may also play outside less (potentially reducing exposure) when it is very hot. Reduced hand contamination in a warmer climate could contribute to reduced diarrhea risk. Diarrhea measurements were outside the scope of this study, but others have observed that *E. coli* presence in water and on hands are associated with diarrhea.^26,108^

This analysis was limited by data availability. The WASH Benefits data were not collected uniformly over the course of each year. As a result, the available data may not be representative of typical seasonal meteorological conditions for the study area. For example, relatively few source water observations were collected at times of year that typically have low precipitation and high temperatures (January, February) and high precipitation and high temperatures (April, May). However, data collection was spread out such that at least some data were collected in every month of the year. Controlling for season or month of data collection was outside of the scope of this analysis. The spatial resolution of the weather data (roughly 30 km^2^ for precipitation and 500 km^2^ for temperature) was also limiting given our study area of 2500 km^2^. Temporal variation in sampling (three-year data collection period) mitigated this. We also did not capture if households stored water in the sun or in the shade. Thus, we may have missed (likely small) differences in temperature and precipitation between nearby households. Finally, we had limited data on handwashing behavior. Field staff observed if respondents washed their hands during the visit; a longer structured observation would have provided richer data and potentially stronger evidence for the presence or absence of relationships between weather and handwashing behavior.

There are important fecal transmission pathways (e.g., fomites, fields, flies, food) that were beyond the scope of this study. Additional work examining the impact of weather on other pathways would be valuable for anticipating climate change impacts and prioritizing interventions. Because mechanisms and behaviors may vary by context, evidence from additional geographic locations would strengthen our understanding of weather impacts on fecal contamination in water and on hands. Because weather has a significant effect on environmental *E. coli* levels, inclusion of weather variables such as temperature and precipitation may improve the precision of estimating intervention effects on fecal contamination in the environment even when weather variables are not the primary exposures of interest. Satellite data enables the incorporation of weather data with relative ease.

We show that heavy precipitation and high temperatures affect water quality and hand contamination levels in rural Kenyan households. Extreme weather due to climate change may increase bacterial contamination in drinking water but reduce contamination on child hands. We suggest that water treatment may be particularly important after periods of heavy precipitation or high temperatures and that climate resiliency efforts should include strategies to make treated water accessible for all.

## Supporting Information Available

Additional details on methods, summary of all hypotheses and statistical tests, and supplementary results (PDF)
